# Determinants of HPV vaccination intentions among Dutch girls and their mothers: a cross-sectional study

**DOI:** 10.1186/1471-2458-13-111

**Published:** 2013-02-06

**Authors:** Hilde M van Keulen, Wilma Otten, Robert AC Ruiter, Minne Fekkes, Jim van Steenbergen, Elise Dusseldorp, Theo WGM Paulussen

**Affiliations:** 1 TNO (Netherlands Organization for Applied Scientific Research), Expertise Center Life Style, PO Box 2215, Leiden, 2301 CE, the Netherlands; 2 Department of Work and Social Psychology, Maastricht University, PO Box 616, Maastricht, 6200 MD, the Netherlands; 3 National Institute for Public Health and the Environment (RIVM), Center for Infectious Disease Control, PO Box 1, Bilthoven, 3720 BA, the Netherlands; 4 Leiden University Medical Center, Center for Infectious Diseases, PO Box 9600, Leiden, 2300 RC, the Netherlands

**Keywords:** HPV, Vaccination intention, Screening, Cancer, Social-psychological determinants

## Abstract

**Background:**

The Dutch government recently added universal Human Papilloma Virus (HPV) vaccination for 12-year-old girls to the existing national immunization program. The participation rate for the initial catch-up campaign for girls aged 13 to 16 years in 2009 was lower (47%) than expected (70%). To inform future HPV information campaigns, this paper examines the social and psychological determinants of the HPV vaccination intentions of girls aged 13 to 16 years and their mothers who were targeted by the Dutch catch-up campaign of 2009.

**Methods:**

A random sample of girls and their mothers was chosen from the Dutch vaccination register and received a letter inviting them to participate (n = 5,998 mothers and daughters). In addition, a random sample was recruited via an online panel by a marketing research company (n = 650 mothers; n = 350 daughters). Both groups were asked to complete a web-based questionnaire with questions on social demographic characteristics, social-psychological factors and HPV vaccination intention. Backward linear regression analyses were conducted to examine which social-psychological factors were most dominantly associated with vaccination intention.

**Results:**

Data from 952 mothers (14%) and 642 daughters (10%) were available for the intended analyses. The contribution of social demographic variables to the explained variance of HPV vaccination intention was small but significant for mothers (ΔR^2^ = .01; *p* = .007), but not significant for daughters (ΔR^2^ = .02; *p* = .17) after controlling for HPV vaccination uptake and the sample. In addition, social-psychological determinants largely contributed to the explained variance of HPV vaccination intention of mothers (ΔR^2^ = .35; *p* < .001) and daughters (ΔR^2^ = .34; *p* < .001). Attitudes, beliefs, subjective norms and habit strength were significantly associated with participants’ HPV vaccination intentions.

**Conclusions:**

Because of the large contribution of social-psychological variables to the explained variance of HPV vaccination intentions among the mothers and daughters, future communication strategies targeting HPV vaccination uptake should address attitudes, beliefs, subjective norms and habit strength. There is a need for longitudinal research to confirm the causality of the association between these determinants and HPV vaccination behavior indicated by this study.

## Background

Despite a long-standing efficient national cervical cancer screening program for women aged 30 to 60 (uptake 66%) [1], 600 new cases of cervical cancer are still diagnosed every year in the Netherlands, and 200 of these patients will eventually die from the disease [2]. The major cause of cervical cancer is persistent infection by the Human Papilloma Virus (HPV) [3], the most common sexually transmitted infection among young women [2,3]. Of all HPV types, types 16 and 18 are responsible for about 70% of cervical cancer cases [4,5]. To prevent persistent HPV infection, two vaccines have been licensed in Europe: Cervarix® GSK and Gardasil®. The Dutch Health Council estimated that the annual number of new cervical cancer cases in the Netherlands could be reduced by 50% by adding universal HPV vaccination of 12-year-old girls to the cervical cancer screening program [2]. The Dutch government decided to implement the Cervarix® GSK vaccine for this age group in the National Immunization Program (NIP), starting in 2010. Each year, the new cohort of 12-year-old girls will be invited to receive the HPV vaccination. The full schedule for this non-mandatory, free vaccine includes three injections (i.e., baseline, at one month and at six months).

In 2009, a HPV vaccination catch-up campaign was organized for girls born between 1993 and 1996 (at that time, 13 to 16 years of age). As with other NIP campaigns, this campaign was coordinated by the National Institute for Public Health and the Environment (RIVM). The accompanying information campaign consisted of an information pamphlet sent to the home addresses of the girls invited, a website with information about the HPV vaccination for girls and parents, and references to a help line. The Community Health Services, which is responsible for the local implementation of the HPV vaccination, organized local mass vaccination sites for girls.

The Dutch NIP, initially implemented in 1999, is one of the most cost-effective public health programs with a consistently high and stable vaccine coverage [6]. About 95% of infants and young children in the Netherlands are vaccinated under the universal childhood vaccination program [7]. The expected participation rate for the HPV campaign was 70%, for several reasons (e.g., the novelty of the vaccine, a new age group, the targeting only of girls and a vaccine targeting sexual transmitted infection) [8]. However, the 2009 participation rate turned out to be much lower, with 57%, 56% and 52% of the invited girls completing one, two, and three vaccinations, respectively [9]. The participation rate remained low in 2010 and 2011 (56% and 54%, respectively) [10,11].

In response to this rather low participation rate, research into the social-psychological determinants of the HPV vaccination decision was initiated to provide direction for the improvement of future HPV vaccine communication to girls and parents used by public health officials to improve vaccination uptake. Because dropout after the first HPV injection (i.e., those who received only one dose) was low in 2010 (5%), these insights into the decision to receive the vaccination can be used to improve the total vaccination uptake. This paper reports on the outcomes of our research on the social-psychological determinants of the HPV vaccination intentions of girls and parents who were targeted by the Dutch catch-up campaign in 2009.

Because the HPV vaccine has only recently been introduced worldwide, most studies have focused on determinants of future acceptance of the HPV vaccination before implementation [12-15]. Reviews indicate that the following factors were most likely to be associated with future acceptance of the HPV vaccine: perceived effectiveness of the vaccine, perceived risks of the vaccine, perceived barriers (e.g., vaccine costs, concerns that the vaccine would promote adolescent sexual behavior), physicians’ recommendations and the opinions of significant others (i.e., subjective norms) [12-15]. Only a few studies have examined determinants of HPV vaccination acceptance after implementation of the vaccine [16-20]. Most of the determinants found in studies conducted before implementation were confirmed empirically by the studies following implementation of the HPV vaccine [16-20]. However, studies conducted after implementation found additional factors: attitude, knowledge, perceived harm and perceived behavioral control [16-20].

Even after implementation of the HPV vaccine, determinants of intention to take the HPV vaccination among girls and parents who do not have to make the decision in the immediate future may differ from those who have actually made the decision and were asked whether they would make it again. Focusing on participants in the latter group is important, because it may improve the explained variance of HPV vaccination intention.

The present study is one of the first to examine determinants of the HPV vaccination intentions among participants who made a decision about the HPV vaccination. In addition, this study is one of the first to examine the extent to which determinants contribute to the HPV vaccination intention. The participants were Dutch girls and their mothers who received an invitation for the first catch-up campaign in 2009.

Given the young age of the girls involved, most studies acknowledge that parents play a large role in their daughters’ HPV vaccination decision [12,16-27]. Some of these studies specifically focused on mothers [16,18,25]. The present study focuses on the HPV vaccination intention of both mothers and girls in order to explore the possible differential impact of the determinants on their HPV vaccination decision making. The research question is as follows: what are the social psychological determinants of the HPV vaccination intentions of girls and their mothers who were targeted by the Dutch catch-up campaign in 2009?

## Methods

This cross-sectional study used a quantitative retrospective approach by means of a web-based questionnaire offered to girls born in 1995 and 1996 (at that time, aged 13 and 14 years) who were invited for the catch-up HPV vaccination campaign in 2009 and their mothers. We recruited study participants among this youngest cohort only, because they best resembled the girls who will be targeted by future campaigns. This research conformed to the Helsinki Declaration and to local legislation. The study was exempt from ethical review according to the Independent Review Board Amsterdam, a medical ethics committee acknowledged by the Central Committee on Research Involving Human Subjects (CCMO).

The girls and their mothers received information about the study in the invitation letter. Those who chose to participate gave their informed consent by filling out the questionnaire. In the invitation letter and at the start of the questionnaire, participants were assured of their privacy, the confidentiality and security of responses, and informed that they could withdraw their participation at any time. Their responses were anonymized; the researchers were unable to verify participants’ names, addresses and e-mail addresses. Participants accessed the online questionnaire by using a log-in code, a unique number. This number was only used by a third party contact person from Praeventis for the Praeventis sample and an online panel bureau for the online panel sample to send reminders to those who did not respond to the first invitation; see “Participants” section for more information about the samples. Participants who completed the questionnaire were not able to participate for a second time.

### Participants

**Figure 1:**
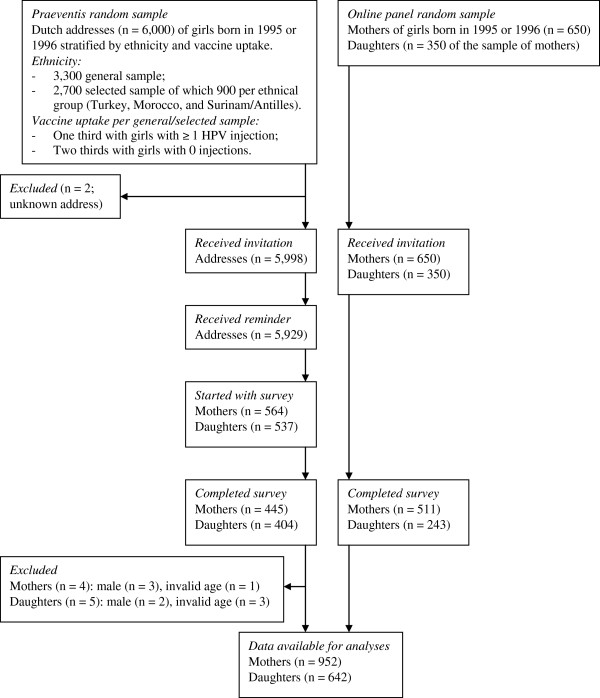
Flow diagram of the recruitment and response of study participants.

A flow diagram of the recruitment and response of participants is presented in Figure 1. The girls and mothers were invited to participate in the study from November 2009 until January 2010. At that time, those girls who agreed to take the HPV vaccination could have received three injections.

#### Praeventis

Participants were invited via Praeventis, the Dutch vaccination register hosted by RIVM. Mothers and daughters that were invited came from the same family unit. A computer program was used to draw a random sample of 6,000 addresses of girls born in 1995 or 1996 that was stratified on known geographical differences in vaccine uptake and ethnicity. Since the addresses of two participants were unknown, the study invitation was sent to 5,998 addresses. The invitation consisted of a single letter addressing mothers of girls born in 1995 or 1996 and the girls themselves. The invitation included a brief description of the study purpose, information about anonymity of participation, a log-in number and an explanation of the log-in procedure.

The online questionnaire for the Praeventis sample was hosted on a private part of the TNO website, which was only accessible through use of the log-in number. Computer assisted self-interviewing was used for the online questionnaire and participants were not rewarded for filling out the questionnaire. The questionnaire was open for response from November 30, 2009, until January 10, 2010. Mothers and daughters (n = 5,929; 99%) who had not responded within three weeks after the first invitation received a reminder. Of the 5,998 invited participants from Praeventis, 564 mothers (9%) and 537 daughters (9%) started the questionnaire, of which 445 mothers (79%) and 404 daughters (75%) fully completed the survey. A large percentage of mothers and daughters who started (21% and 25%, respectively) failed to complete the questionnaire. Male participants (n = 5) and those with an invalid age (n = 4) were excluded from the analyses. The final response rate was 7% (N = 441) among the mothers, and 7% (N = 399) among the daughters.

#### Online panel

Due to the Praeventis sample’s limited response to the first invitation letter, we decided to recruit an extra sample by using an online panel of a private marketing research company. A computer program was used to draw a random sample of 650 mothers of girls born in 1995 or 1996, and 350 of their daughters that was stratified on age, education and geographical differences in vaccine uptake. Among the online panel sample, 350 invited mothers and daughters came from the same family unit. They were sent an e-mail that offered the opportunity to complete the questionnaire between December 18 and December 24, 2009. The online panel was responsible for collecting the data among the sample and TNO was owner of the data. Computer assisted self-interviewing was used for the online questionnaire, which was hosted on a private part of the online panel website that was only accessible through use of the log-in number. Participants from the online panel sample were rewarded for completing the questionnaire according to the standards of the online panel company: they received panel points that could be exchanged for gift coupons. The points that each participant received for completing the questionnaire about HPV vaccination were worth 4.35 euros. The response rate of people contacted by this recruitment strategy was 79% (N = 511) among the mothers, and 69% (N = 243) among the daughters.

In total, data from 952 mothers (14%) and 642 of their daughters (10%) were available for the intended analyses. The total sample included 497 mother-daughter pairs, and 455 and 145 independent mothers and daughters, respectively.

### Questionnaire

The online questionnaire was based on information derived from former empirical research on general vaccination intention, HPV vaccination intention (see Table 1 for references) and qualitative focus groups with representatives from the study population. The Theory of Planned Behavior [28], Social Cognitive Theory [29] and the Health Belief Model [30] formed the theoretical framework of this study. Mothers received questions about the HPV vaccination of their daughter, whereas daughters answered questions about the HPV vaccination of themselves. The questionnaire was pretested on a small sample from the study population, and subsequently revised. The revised questionnaire is described below.

**Table 1:** **Overview of social-psychological scale measures**^**1**^

**Measure**	**Item**	**Answer options**	**Scale (minimum to maximum value)**	**Number of items**	**Cronbach’s alpha (α)**	**Reference**
Knowledge about the HPV vaccination^a^	־ HPV is sexually transmittable;	−1 = incorrect	−8 = incorrect	8	n/a	
	־ HPV is a virus;	0 = don’t know	8 = correct			
	־ The HPV vaccination in the Netherlands consists of three injections;	1 = correct				
	־ My daughter is/I am obliged to get the HPV vaccination when she is/I am invited;					
	־ You will always notice when you are infected by HPV;					
	־ Only women can get infected by HPV;					
	־ Women who received the HPV vaccination are still advised to participate in the cervical cancer screening in the Netherlands;					
	־ The HPV vaccination fully protects against cervical cancer.					
Risk perception (having received no HPV vaccination)	Imagine that your daughter was/you were not vaccinated against HPV. The chance that my daughter will get/I will get cervical cancer is…	−3 = very small to 3 = very large	n/a	1	n/a	[20,31]
Risk perception (having received the HPV vaccination)	Imagine that your daughter was/you were vaccinated against HPV. The chance that my daughter will get/I will get cervical cancer is…	−3 = very small to 3 = very large	n/a	1	n/a	[20,31]
Attitude towards the HPV vaccination	Vaccinating my daughter/myself against HPV is…	−3 = very undesirable to 3 = very desirable;	−12 = negative to 12 = positive	4	M = .98	[31]
					D = .94	
		−3 = very bad to 3 = very good;				
		−3 = very negative to 3 = very positive;				
		−3 = very unimportant to 3 = very important				
Negative outcome expectations of the HPV vaccination	If my daughter gets/I get the HPV vaccination…	−3 = completely agree to 3 = completely disagree	−15 = completely agree to 15 = completely disagree	5	M = .60	[20,27,32]
					D = .49	
	־ she/I will become infertile;					
	־ she/I will get unpleasant side effects shortly after the injection, such as head ache, fever or pain at the injection spot;					
	־ she/I will be afraid of the injection with a needle;					
	־ she/I will have unsafe sex in the future;					
	־ her/my natural immune system against illnesses will be disturbed.					
Positive outcome expectations of the HPV vaccination	If my daughter gets/I get the HPV vaccination…	−3 = completely disagree to 3 = completely agree	−9 = completely disagree to 9 = completely agree	3	M = .76	[33,34]
					D = .77	
	־ she/I will not contract cervical cancer;					
	־ she/I will not have to worry about cervical cancer;					
	־ she/I will not be infected with HPV.					
Anticipated regret about rejecting the HPV vaccination	Imagine your daughter has not received the HPV vaccination and she gets cervical cancer in the future. How much would you regret your decision to let her receive no vaccination?	1 = no regret and 5 = very much regret	n/a	1	n/a	
Anticipated regret about receiving the HPV vaccination	Imagine your daughter has received the HPV vaccination and she gets a serious illness as a result of the vaccine. How much would you regret your decision to let her receive the vaccination?	1 = no regret and 5 = very much regret	n/a	1	n/a	
Beliefs about the HPV vaccination	־ If the government offers the vaccination, I assume it will be safe;	−3 = completely disagree to 3 = completely agree	−18 = negative to 18 = positive	9	M = .80	[19,20]
					D = .70	
	־ Our government shows responsibility for the health of the Dutch population by introducing the HPV vaccination;	−3 = completely agree to 3 = completely disagree				
	־ The HPV vaccination was only introduced because the pharmaceutical industry will earn a lot of money from it;					
	־ There is too little known about whether					
	the HPV vaccination effectively protects against cervical cancer;					
	־ There is too little known about the detrimental side effects of the HPV vaccination;					
	־ My daughter is/I am too young to receive the HPV vaccination;					
	־ My daughter does/I do not need to get the HPV vaccination if she is/I am already sexually active;					
	־ My daughter does/I do not need the vaccination because she is/I am not yet sexually active;					
	־ It is inappropriate that my daughter has/I have to pay for the HPV vaccination if she decides/I decide to get the vaccination at a higher age.					
Relative effectiveness of the HPV vaccination^b^	How would you rate the effectiveness of the following methods of preventing cervical cancer:	1 = not at all effective to 10 = very effective	n/a	5	n/a	

	־ having safe sex					
	־ having sex with only one person in a lifetime					
	־ participating in the cervical cancer screening					
	־ having a healthy lifestyle (e.g. not smoking)					
	־ the HPV vaccination					
	Participants rated the effectiveness of each method					
Ambivalence towards the HPV vaccination decision	During my decision making about my daughter’s/my HPV vaccination…	−3 = completely disagree to 3 = completely agree	−9 = completely disagree to 9 = completely agree	3	M = .93	
					D = .85	
	־ I felt torn between the pros and cons of vaccination;					
	־ I experienced positive as well as negative feelings;					
	־ I was torn between the pros and cons of vaccination.					
Confidence in authorities involved in the HPV vaccination	As regards the HPV vaccination, how much confidence do you have in…	−3 = very little to 3 = very much confidence	−9 = very little to 9 = very much confidence	3	M = .91	
					D = .88	
	־ science					
	־ health care					
	־ the Ministry of Public Health					
	Participants rated their confidence in each authority					
Subjective norms towards the HPV vaccination^c^	*Normative beliefs*	−2 = certainly not vaccinating to 2 = certainly vaccinating,	−70 =negative to 70 = positive (M);	M = 7	M = .85	[31]
	What is your expectation on the opinion of … about the HPV vaccination of your daughter?			D = 6	D = .77	
		3 = not applicable;	−60 =negative to 60 = positive (D)			
		Not applicable was recoded into ‘0’				
	Social referents: partner (M), parents (M), daughter (M), father (D), mother (D), best friends (M/D), general practitioner (M/D), doctor/nurse from the municipal health service (M/D), the Ministry of Public Health (M/D)					
	*Motivation to comply*	1 = not at all to 5 = very much		M = 7		[31]
	How motivated are you to comply with the opinion of …?			D = 6		
Descriptive norms towards the HPV vaccination	How many parents/girls do you know who will decide to let their daughter/themselves be vaccinated against HPV if they receive the invitation?	1 = none of the parents/girls I know, to 7 = all the parents/girls I know	n/a	1	n/a	[31]
Self-efficacy expectations towards the HPV vaccination	To what extent would you succeed in dealing with the following situations:	−3 = I would certainly not succeed to 3 = I would certainly succeed	−18 = I would certainly not succeed to 18 = I would certainly succeed	6	M = .82	
					D = .66	
	־ Finding reliable information about the HPV vaccination;					
	־ Understanding information about the HPV vaccination;					
	־ Making a different decision about the HPV vaccination of my daughter/myself than most parents/girls among my acquaintances;					
	־ Having a good talk with my daughter about the HPV vaccination (M);					
	־ Having a good talk with my partner about the HPV vaccination (M);					
	־ Having a good talk with my father about the HPV vaccination (D);					
	־ Having a good talk with my mother about the HPV vaccination (D);					
	־ Having a good talk with my general practitioner about the HPV vaccination.					
HPV vaccination information processing	Before I finally decided about my (daughter’s) vaccination, I…		−6 = completely disagree to 6 = completely agree	2	M = .84	[31]
					D = .75	
	־ gathered a lot of information about the HPV vaccine					
	־ thoroughly considered the HPV vaccination	−3 = completely disagree to 3 = completely agree				
	Participants rated their agreement on both items separately					
Habit strength towards the HPV vaccination	Letting my daughter receive the HPV vaccination/Receiving the HPV vaccination is something I do…	−3 = completely disagree to 3 = completely agree	−6 = completely disagree to 6 = completely agree	2	M = .78	[35]
					D = .65	
	־ automatically					
	־ without thinking					
	Participants rated their agreement on both items separately					
Decisional conflict about the HPV vaccination - value clarity	As regards the HPV vaccination…	−3 = completely disagree to 3 = completely agree	−9 = completely disagree to 9 = completely agree	3	M = .89	[36]

	־ I was clear about which benefits were most important to me;				D = .85	
	־ I was clear about which risks were most important to me;					
	־ I was clear about which was more important to me, the benefits or the risks.					
Decisional conflict about the HPV vaccination - certainty	As regards the HPV vaccination…	−3 = completely disagree to 3 = completely agree	−9 = completely disagree to 9 = completely agree	3	M = .89	[36]
					D = .87	
	־ I felt sure about what to choose;					
	־ the decision was relatively easy to make;					
	־ I was clear about the best choice for me/my daughter.					
Opinion about alternative medicine	In what way do you agree with the view of…	−3 = completely disagree to 3 = completely agree	−9 = completely disagree to 9 = completely agree	3	M = .81	
	־ anthroposophy				D = .76	
	־ homeopathy					
	־ other alternative medicine					
Past cancer experience^d^	Have you/has your mother had experience with…	1 = no, 2 = yes, 3 = I do not want to answer this question	n/a	6	n/a	[19,27]
	Do you know someone from your close environment who has/has had…					
	־ cervical cancer					
	־ other cancers					
	־ abnormal pap smear					

*Notes* M = Mother; D = Daughter; n/a = not applicable; 1Scores on scaled item that showed sufficient internal consistency were summed into one scale; Mothers received questions about the HPV vaccination of their daughter, whereas daughters answered questions about the HPV vaccination of themselves; a) Knowledge is not a scale because the answer on one item does not predict the answer on the other items; the items were summed up to present a sum score of knowledge; b) The difference between the rated effectiveness of the HPV vaccination and the most effective alternative represented the relative effectiveness score (−9 = HPV vaccination least effective to 9 = HPV vaccination most effective); c) The subjective norms score was first computed by multiplying normative beliefs and motivation to comply for each social referent, and then by summing up the multiplications of the social referents; d) Past cancer experience is not a scale because the answer on one item does not predict the answer on the other items.

#### Vaccination intention

Because social-psychological factors were measured at present and vaccination intention refers to future behavior, vaccination intention rather than past vaccination behavior was used as the criterion variable for examining the relationship between social-psychological factors and the HPV vaccination decision. Intention is a powerful predictor of infrequently performed behavior, such as obtaining a vaccination [37]. Vaccination intention was assessed by asking “If you had to make the HPV vaccination decision again, would you vaccinate your daughter/yourself against HPV?” (−3 = certainly not, to 3 = certainly yes) [31].

#### Vaccination uptake

Vaccination uptake was assessed by a dichotomous variable representing complete vaccination (no = received less than three HPV injections; yes = received all three HPV injections)*.* For participants recruited via Praeventis, HPV vaccination uptake information was available via the Praeventis vaccine register (i.e., number of HPV injections) as well as the survey (i.e., “Has your daughter received/Did you receive all three HPV injections?” (yes/no)). The percentage of agreement between objective and self-reported vaccination uptake for mothers and daughters from the Praeventis sample was determined by comparing the association between the Praeventis registry information with responses in the questionnaire. For participants recruited by the online panel, HPV vaccination uptake was available only via the survey (“How many HPV injections did your daughter/you receive?” (0 to 3 injections)).

#### Social-psychological factors

Social-psychological factors included knowledge, risk perception, attitude, outcome expectations, anticipated regret, beliefs, perceived relative effectiveness of the HPV vaccination, ambivalence, confidence in responsible authorities, subjective norms, descriptive norms, self-efficacy expectations, information processing, habit, decisional conflict (certainty, and value clarity), opinions about alternative medicine and past cancer experience (see Table 1 for an overview of the measures, sample items and the Cronbach’s alpha). Scores on items that, taken as a group, had meaningful content coverage [38] and showed sufficient internal consistency (Cronbach’s alpha > 0.60 in at least one of the two samples) were summed into one scale. With regard to risk perception (i.e., perceived susceptibility and perceived severity), perceived severity was not measured because low variability was expected and a recent review showed no association with vaccine intention [12].

#### Socio-demographic factors

Socio-demographic factors were included to explore the need for segmentation of target groups in future educational interventions. The study accounted for age, gender, educational level, country of birth and religion.

*Level of education* was measured by asking mothers about the highest level of education that they had completed and by asking daughters about their own education and the highest level of education both their parents had completed (the parent with the highest completed educational level was used to rank the educational level of the parents). Highest completed educational level for adults was classified as low (less than secondary or vocational education), intermediate (secondary through pre-university education) or high (professional or university education). For the daughters, attained educational level was classified as intermediate (senior general secondary education or pre-university education) or low (other).

*Country of birth* was also assessed for mothers and daughters. The variables were classified into four categories which represent the largest ethnic groups in the Netherlands and matched our recruitment strategy: Netherlands, Surinam/Antilles/Aruba, Turkey/Morocco and other countries.

*Religion* was measured by asking participants about their religious convictions (Protestant, Roman Catholic, Muslim, Jewish, Buddhist, Hindu, other or no religion). Because a small minority of participants had another religion than Roman Catholic or Protestant, these participants were classified as “Roman Catholic” and people with “no religion” were put in the category “no religion”. This information was further dichotomized into “Protestant” or “not Protestant” because an ANOVA revealed that Protestant participants had a significantly lower vaccine intention than Roman Catholics or people with no religion. Finally, we asked participants if the parents had differences in opinion about the acceptability of the HPV vaccine.

### Data analyses

In all analyses, education level was treated as nominal, instead of ordinal, because we did not assume a monotonic increasing relationship of education level with the outcome variable “HPV-vaccination intention”. The association between objective and self-reported vaccination uptake (i.e., received all three HPV injections versus received less than three HPV injections) for mothers and daughters was determined by chi-square tests. Differences between participants recruited from Praeventis and the online panel were examined by t-tests or chi-square tests with Statistical Package for Social Sciences (SPSS) 17.0. A two-sided alpha of .05 was used as the criterion for significance.

HPV vaccination intention was used as the criterion variable for examining the relationship between social-psychological factors and the HPV vaccination decision, as it is consistent with behavioral theories (e.g., Theory of Planned Behavior) that explain future behavior via intention, by social-psychological determinants measured at present. We first examined the univariate relationship between social-psychological factors and vaccination intention with simple regression analysis. Because of the large amount of univariate tests, we used a two-sided alpha of .01 as the criterion for significance. Secondly, we conducted a backward linear regression analysis to examine which social-psychological factors were most dominantly associated with vaccination intention. Only significant factors from the univariate tests were included in the regression analyses as predictors in a fourth step, after correcting for HPV vaccination uptake in the first step, sample in the second step and socio-demographic variables in a third step. HPV vaccination uptake was included in the regression analysis as a first step because past behavior best predicts future behavior [28]. A two-sided *p* ≥ .01 was used as the criterion for removal of a predictor in the backwards selection procedure. HPV vaccination uptake, sample, socio-demographic and social-psychological factors were included in the model by the forced entry method to determine their unique explained variance on vaccination intention.

To select the most important factors, the backward method was used to exclude non-significant variables (two-sided *p* ≥ .01) from the model, except for HPV vaccination uptake, sample and socio-demographic variables for which differences were found between the two samples. Because we wanted to adjust for the latter variables in the final model, we applied a manually backward selection, instead of an automatic procedure. There was no indication of multicollinearity between variables in the regression model (variance inflation factor values < 10). To further shape future recommendations, Pearson’s correlation coefficients were calculated for the relationship between vaccination intention and individual study measure items that were significantly related to vaccination intention according to the multiple regression model.

The univariate and multivariate tests were first performed on a random sample of 75% of the participants. Data from the other (25%) participants were used to check for stability and generalizability. The stability check examined which predictors from the final regression model among the 75% sample remained significant in the 25% sample; these were expected to be stable predictors. The generalizability check examined the predictive value of the final regression models for the vaccination intention of the population. This check was conducted to account for overestimation of the percentage of explained variance which mostly occurs in a regression analysis. The goodness-of-fit of the final regression model for the 75% sample was estimated for the 25% sample by keeping the estimated parameters (Betas) of the regression model for the 75% sample. This analysis was performed in R [39]. Subsequently, the percentage of explained variance was compared between the two samples, with large differences indicating a large amount of overfitting [40].

## Results

### Sample

#### Sample description

In total, we analyzed data from 952 mothers and 642 daughters. No data were available from participants who did not respond to the invitation. The sample description is depicted in Table 2.

**Table 2:** **Sample description (percentage or mean** ± **standard deviation)**^**1**^

**Variables**	**Mothers**			**Daughters**		
	**Praeventis (n=441)**	**Panel (n=511)**	**Total (n=952)**	**Praeventis (n=399)**	**Panel (n=243)**	**Total (n=642)**
Age	43.81 ± 4.74	43.01 ± 4.48	43.38 ± 4.62*	13.52 ± 0.51	13.50 ± 0.51	13.51 ± 0.51
Educational level mother	N_missing_ = 2		N_missing_ = 2*	-	-	-
Low	24%	27%	26%			
Intermediate	39%	49%	44%			
High	37%	24%	30%			
Educational level parents	-	-	-	N_missing_ = 19	N_missing_ = 19	N_missing_ = 38*
Low				26%	17%	22%
Intermediate				34%	26%	31%
High				40%	57%	46%
Educational level daughter	-	-	-		n=238	n=637*
Low				43%	84%	58%
Intermediate				57%	16%	42%
Country of birth mother	N_missing_ = 1	N_missing_ = 3	N_missing_ = 4*	N_missing_ = 10	N_missing_ = 20	N_missing_ = 30*
The Netherlands	77%	97%	88%	63%	96%	75%
Surinam/Antilles	8%	1%	4%	11%	1%	7%
Turkey/Morocco	10%	0%	5%	21%	0%	13%
Other	5%	2%	3%	5%	3%	4%
Country of birth daughter	-	-	-			*
The Netherlands				90%	98%	93%
Surinam/Antilles				3%	0%	2%
Turkey/Morocco				4%	0%	2%
Other				3%	2%	3%
Religion	N_missing_ = 2	N_missing_ = 1	N_missing_ = 3*			*
Protestant	18%	29%	24%	15%	22%	18%
Number of HPV injections^2^	1.42 ± 1.45	1.59 ± 1.42	1.51 ± 1.44	1.47 ± 1.46	1.72 ± 1.44	1.56 ± 1.46
None	50%	43%	46%	49%	40%	46%
One	1%	1%	1%	1%	1%	1%
Two	6%	10%	8%	5%	6%	5%
Three	43%	46%	45%	45%	53%	48%
Three HPV injections received (self-reported)	45%	46%	45%	49%	53%	51%
HPV vaccination intention	0.57 ± 2.07	0.57 ± 2.20	0.57 ± 2.14	0.43 ± 1.98	0.61 ± 2.03	0.50 ± 2.00
Certainly not	12%	14%	13%	11%	10%	11%
Not	8%	12%	10%	9%	10%	9%
Probably not	12%	9%	11%	13%	11%	12%
Probably not/yes	11%	7%	9%	15%	14%	15%
Probably yes	14%	10%	12%	16%	12%	14%
Yes	19%	22%	20%	17%	18%	17%
Certainly yes	24%	26%	25%	20%	25%	22%

*Notes* 1) In case of missing values, the number of missing values (Nmissing) were presented; Mothers received questions about the HPV vaccination of their daughter, whereas daughters answered questions about the HPV vaccination of themselves; 2) These number represents daughters and are obtained from the vaccine registration for participants recruited via Praeventis, and are self-reported for participants recruited via an online panel; *Significant difference between Praeventis and the panel (*p* < .05).

#### Socio-demographic factors

The mean age of the mothers and daughters was 43 years (SD = 4.6) and 13 years (SD = 0.5), respectively. Most of the mothers (88%) and daughters (75%) were born in the Netherlands, and 24% and 18% were Protestant, respectively. One quarter of the mothers (26%) had a low educational level. More than half of the girls (58%) attained a low educational level. The sample appeared to represent the general population in the Netherlands with regard to educational level [41], country of birth [42] and religion [43]. A small percentage (5%) of mothers who had a partner (n = 880) indicated that they differed in opinion from their partner about the HPV vaccination. Eight percent of the daughters who had two parents (n = 595) indicated that their parents differed in opinion about the HPV vaccination.

#### Vaccination intention

Less than half of the mothers (45%) and daughters (39%) indicated that they would like or certainly like to receive the HPV vaccination if they were to decide in the future (Table 2). Among the mother-daughter pairs (n = 497), the vaccination intention of mothers strongly correlated with that of daughters (*r* = .77; *p* < .001).

#### Vaccination uptake

Almost half of the mothers (45%) indicated that their daughter received all three HPV injections in 2009, and 51% of the daughters indicated that they had received them. The association between objective and self-reported data on the number of received HPV injections (“has not received all three injections” versus “has received all three injections”) in the Praeventis sample was high (mothers: n = 441; *χ*^2^ = 290.56; p < .001; daughters: n = 399; *χ*^2^ = 325.02; p < .001), and the percentage of agreement between both outcome variables appeared very large (91% for mothers and 94% for daughters). The correlation between HPV vaccination uptake and future vaccination intention was also significant for mothers (*r* = .69; *p* < .001) and daughters (*r* = .62; *p* < .001).

#### *Differences between participants recruited* via *Praeventis and the online panel*

Compared to mothers recruited via the online panel, those recruited via Praeventis were older (online panel 43.0 versus Praeventis 43.8 years of age; t = 2.69, *p* = .007, Table 2) and had completed a higher level of education (24% versus 37%; *χ*^2^ = 17.73, *p* < .001), fewer mothers were born in the Netherlands (97% versus 77%; *χ*^2^ = 94.23, *p* < .001) and fewer mothers were Protestant (29% versus 18%; *χ*^2^ = 13.43, *p* < .001). Among mothers, there were no differences between the samples with regard to HPV vaccination intention (*p* = .95) and vaccination uptake (*p* = .68).

Compared to daughters recruited via the online panel, those recruited via Praeventis were more likely to have parents with a lower completed level of education (17% versus 26%; *χ*^2^ = 17.14, *p* < .001), were less likely to attain a lower educational level themselves (84% versus 43%; *χ*^2^ = 103.93, *p* < .001), were less likely to have a mother who was born in the Netherlands (96% versus 63%; *χ*^2^ = 90.66, *p* < .001), were less likely to have been born in the Netherlands themselves (98% versus 90%; *χ*^2^ = 17.97, *p* < .001) and were less likely to be Protestant (22% versus 15%; *χ*^2^ = 4.94, *p* = .03). Among daughters, there were no differences between the samples with regard to age (*p* = .73), HPV vaccination intention (*p* = .28) and vaccination uptake (*p* = .36).

### Relationship between determinants and the HPV vaccination intention

#### Univariate tests

According to the univariate regression analyses, the following factors significantly contributed to the mothers’ (Table 3) and daughters’ (Table 4) HPV vaccination intention: HPV vaccination uptake, religion, risk perception without having received the HPV vaccination, attitude, positive and negative outcome expectations, anticipated regret with and without having received the HPV vaccination, beliefs, relative effectiveness of the HPV vaccination, confidence in responsible authorities, subjective norms, descriptive norms, information processing and habit strength. Among mothers, significance was also found for ambivalence, decisional conflict (certainty) and opinion about alternative medicine; among daughters, significance was also found for self-efficacy and decisional conflict (values clarity).

**Table 3:** Relationship of socio-demographic variables and social-psychological factors with the mothers’ HPV vaccination intention

**Variables**	**Univariate simple regression analysis among 75% of the mothers (n = 732)**		**Multivariate backward regression analysis among 75% of the mothers (n = 727)**		**Multivariate backward regression analysis among 25% of the mothers (n = 219)**	
	**Mean (standard deviation) or percentage**	**Association**^**1**^	**Beta (standard error)**	**Standardized Beta**	**Beta (standard error)**	**Standardized Beta**
*HPV vaccination uptake*		.48**	0.48 (0.10)	.11**	0.57 (0.19)	.13*
Has not received three HPV-injections (reference)	54%					
Has received three HPV-injections	46%					
						
*Sample*		.00	−0.01 (0.07)	0.00	0.15 (0.14)	.04
Praeventis (reference)	47%					
Online panel	53%					
						
*Socio-demographic variables*						
Age	43.51 (4.54)	.00	−0.01 (0.01)	-.02	0.00 (0.01)	.01
Highest completed level of education mother		.00				
Low (reference)	25%					
Intermediate	45%		0.08 (0.08)	.02	−0.02 (0.17)	-.00
High	30%		0.13 (0.09)	.03	0.27 (0.18)	.06
Country of birth mother		.00				
The Netherlands (reference)	88%					
Surinam/Antilles	4%		−0.22 (0.18)	-.02	0.42 (0.32)	.04
Turkey/Morocco	4%		−0.51 (0.18)	-.05*	0.18 (0.32)	.02
Other	3%		−0.30 (0.18)	-.03	0.10 (0.41)	.01
Protestant religion		.02**				
No (reference)	77%					
Yes	23%		−0.08 (0.08)	-.02	−0.05 (0.15)	-.01
						
*Social psychological variables*						
Knowledge about the HPV vaccination	4.82 (2.42)	.00				
Risk perception (having received no HPV vaccination)	−0.61 (1.21)	.20**				
Risk perception (having received the HPV vaccination)	−1.22 (1.09)	.01				
Attitude towards the HPV vaccination	2.78 (6.93)	.76**	0.12 (0.01)	.39**	0.13 (0.02)	.42**
Negative outcome expectations of the HPV vaccination	7.24 (4.56)	.14**				
Positive outcome expectations of the HPV vaccination	−3.44 (3.86)	.10**				
Anticipated regret about rejecting the HPV vaccination	3.27 (1.44)	.46**	0.20 (0.03)	.13**		
Anticipated regret about receiving the vaccination	3.38 (1.39)	.06**	−0.08 (0.03)	-.05*		
Beliefs about the HPV vaccination	1.42 (9.92)	.60**	0.03 (0.01)	.14**	0.03 (0.01)	.12*
Relative effectiveness of the HPV vaccination	−0.61 (1.20)	.54**				
Ambivalence towards the HPV vaccination decision	0.36 (5.30)	.01*				
Confidence in authorities involved in the HPV vaccination	1.60 (3.49)	.42**				
Subjective norms towards the HPV vaccination	16.81 (23.57)	.67**	0.02 (0.00)	.19**	0.02 (0.01)	.20**
Descriptive norms towards the HPV vaccination	4.53 (1.34)	.15**				
Self-efficacy expectations towards the HPV vaccination	11.87 (4.66)	.01				
HPV vaccination information processing	3.79 (2.40)	.06**				
Habit strength towards the HPV vaccination	−0.81 (3.64)	.41**	0.07 (0.02)	.11**	0.12 (0.03)	.19**
Decisional conflict about the HPV vaccination – value clarity	3.03 (4.29)	.00				
Decisional conflict about the HPV vaccination – certainty	2.00 (4.69)	.02**	−0.05 (.01)	-.10**	−0.07 (0.02)	-.15**
Opinion about alternative medicine	−0.43 (2.37)	.02*				
Past experience of the mother with cervical cancer		.00				
No (reference)	98%					
Yes	2%					
Past experience of the mother with other cancer forms		.00				
No (reference)	97%					
Yes	3%					
Past experience of the mother with abnormal pap smear		.00				
No (reference)	87%					
Yes	13%					
Past experience of someone from the close environment with cervical cancer		.00				
No (reference)	67%					
Yes	33%					
Past experience of someone from the close environment with other cancer forms		.00				
No (reference)	10%					
Yes	90%					
Past experience of someone from the close environment with abnormal pap smear		0.00				
No (reference)	50%					
Yes	50%					
						
*Model fit for multivariate models*						
R^2^ of HPV vaccination uptake				.47		.48
R^2^ change of HPV vaccination uptake				.47		.48
F change of HPV vaccination uptake				653.97**		198.23**
R^2^ of HPV vaccination uptake + sample				.47		.48
R^2^ change of HPV vaccination uptake + sample				.00		.00
F change of HPV vaccination uptake + sample				0.04		0.36
R^2^ of HPV vaccination uptake + sample + socio-demographic variables				.49		.50
R^2^ change of HPV vaccination uptake + sample + socio-demographic variables				.01		.02
F change of HPV vaccination uptake + sample + socio-demographic variables				2.82*		1.37
R^2^ of HPV vaccination uptake + sample + socio-demographic + social-psychological variables				.84		.81
R^2^ change of HPV vaccination uptake + sample + socio-demographic + social-psychological variables				.35		.31
F change of HPV vaccination uptake + sample + socio-demographic + social-psychological variables				218.92**		66.04**

*Notes* 1) R**2; Mothers received questions about the HPV vaccination of their daughter, whereas daughters answered questions about the HPV vaccination of themselves; *p < .01; **p < .001**.**

**Table 4:** Relationship of socio-demographic variables and social-psychological factors with the daughters’ HPV vaccination intention

**Variables**	**Univariate simple regression analysis among 75% of the daughters (n = 482)**		**Multivariate backward regression analysis among 75% of the daughters (n = 452)**		**Multivariate backward regression analysis among 25% of the daughters (n = 147)**	
	**Mean (standard deviation) or percentage**	**Association**^**1**^	**Beta (standard error)**	**Standardized Beta**	**Beta (standard error)**	**Standardized Beta**
*HPV vaccination* uptake		.35**	0.36 (0.14)	.09*	0.92 (0.25)	.23**
Has not received three HPV-injections (reference)	52					
Has received three HPV-injections	48					
						
*Sample*		.00	0.15 (0.13)	.04	0.11 (0.23)	.03
Praeventis (reference)	64					
Online panel	36					
						
*Socio-demographic variables*						
Age	13.51 (0.51)	.00				
Educational level daughter		.00				
Low (reference)	59%					
Intermediate	41%		0.22 (0.12)	.06	−0.11 (0.21)	-.03
Highest completed level of education of parents		.00				
Low (reference)	22%					
Intermediate	32%		0.08 (0.15)	.02	0.18 (0.24)	.04
High	46%		0.04 (0.15)	.01	0.31 (0.23)	.08
Country of birth mother		.00				
The Netherlands (reference)	74%					
Surinam/Antilles	7%		0.05 (0.24)	.01	0.08 (0.41)	.01
Turkey/Morocco	15%		−0.09 (0.19)	-.02	0.25 (0.34)	.04
Other	4%		0.08 (0.26)	.01	−1.18 (0.45)	-.13
Country of birth daughter		.00				
The Netherlands (reference)	92%					
Surinam/Antilles	2%		−0.74 (0.43)	-.05	−0.03 (0.59)	−0.00
Turkey/Morocco	3%		0.21 (0.33)	.02	-	-
Other	3%		0.35 (0.33)	.03	1.62 (0.56)	.13*
Protestant religion		.02*				
No (reference)	82%					
Yes	19%		0.02 (0.13)	.00	−0.15 (0.23)	-.03
*Social psychological variables*						
Knowledge about the HPV vaccination	3.59 (2.41)	.00				
Risk perception (having received no HPV vaccination)	−0.61 (1.39)	.23**	0.19 (0.04)	.13**		
Risk perception (having received the HPV vaccination)	−1.49 (1.22)	.00				
Attitude towards the HPV vaccination	2.43 (6.02)	.62**	0.12 (0.01)	.37**	0.14 (0.03)	.39**
Negative outcome expectations of the HPV vaccination	5.66 (4.71)	.09**				
Positive outcome expectations of the HPV vaccination	−1.02 (4.17)	.10**				
Anticipated regret about rejecting the HPV vaccination	3.24 (1.43)	.27**			0.24 (0.08)	.17*
Anticipated regret about receiving the vaccination	3.11 (1.52)	.09**				
Beliefs about the HPV vaccination	2.11 (8.31)	.47**	0.05 (0.01)	.21**		
Relative effectiveness of the HPV vaccination	−2.45 (2.61)	.36**				
Ambivalence towards the HPV vaccination decision	−0.11 (4.64)	.00				
Confidence in authorities involved in the HPV vaccination	1.46 (3.49)	.29**				
Subjective norms towards the HPV vaccination	13.87 (18.60)	.46**	0.02 (0.00)	.15**	0.03 (0.01)	.23*
Descriptive norms towards the HPV vaccination	4.51 (1.42)	.11**				
Self-efficacy expectations towards the HPV vaccination	1.43 (4.70)	.06**				
HPV vaccination information processing	1.00 (3.16)	.03**				
Habit strength towards the HPV vaccination	−0.28 (3.02)	.32**	0.08 (0.02)	.12**		
Decisional conflict about the HPV vaccination – value clarity	1.92 (3.99)	.02*				
Decisional conflict about the HPV vaccination – certainty	2.06 (4.12)	.01				
Opinion about alternative medicine	−0.28 (1.65)	.00				
Past experience of the mother with cervical cancer		.00				
No (reference)	98%					
Yes	2%					
Past experience of the mother with other cancer forms		.00				
No (reference)	96%					
Yes	4%					
Past experience of the mother with abnormal pap smear		.00				
No (reference)	92%					
Yes	8%					
Past experience of someone from the close environment with cervical cancer		.00				
No (reference)	87%					
Yes	13%					
Past experience of someone from the close environment with other cancer forms		.00				
No (reference)	22%					
Yes	78%					
Past experience of someone from the close environment with abnormal pap smear		.01				
No (reference)	86%					
Yes	14%					
						
*Model fit for multivariate models*						
R^2^ of HPV vaccination uptake				.36		.49
R^2^ change of HPV vaccination uptake				.36		.49
F change of HPV vaccination uptake				257.28**		137.34**
R^2^ of HPV vaccination uptake + sample				.36		.49
R^2^ change of HPV vaccination uptake + sample				.00		.00
F change of HPV vaccination uptake + sample				0.16		0.58
R^2^ of HPV vaccination uptake + sample + socio-demographic variables				.38		.54
R^2^ change of HPV vaccination uptake + sample + socio-demographic variables				.02		.05
F change of HPV vaccination uptake + sample + socio-demographic variables				1.41		1.54
R^2^ of HPV vaccination uptake + sample + socio-demographic + social-psychological variables				.73		.77
R^2^ change of HPV vaccination uptake + sample + socio-demographic + social-psychological variables				.34		.24
F change of HPV vaccination uptake + sample + socio-demographic + social-psychological variables				109.17**		45.91**

*Notes* 1) R**2; Mothers received questions about the HPV vaccination of their daughter, whereas daughters answered questions about the HPV vaccination of themselves; *p < .01; **p < .001**.**

#### Multivariate tests

Tables 3 (mothers) and 4 (daughters) present factors that significantly contributed to the HPV vaccination intention according to the multivariate regression analyses.

#### *Mothers*

The unique contribution of HPV vaccination uptake (first step) to the explained variance of vaccination intention was large and significant (R^2^ = .47, *p* < .001). In addition, the samples (second step) did not significantly contribute to the explained variance of vaccination intention (ΔR^2^ = .00; *p* = .83). The additional contribution of socio-demographic variables (third step) to the explained variance of vaccination intention was small but significant (ΔR^2^ = .01, *p* = .007) for country of birth. The multivariate relationship between country of birth and intention was not found by the univariate test, which was most likely caused by the small percentage of mothers born in Turkey or Morocco (4%). The social-psychological determinants (fourth step) largely and significantly contributed to the explained variance of vaccination intention (ΔR^2^ = .35; *p* < .001) after correcting for HPV vaccination uptake, sample and socio-demographic variables: attitude, anticipated regret when accepting or refraining from vaccination, beliefs, subjective norms, habit strength and decisional conflict (certainty). The relationship between decisional conflict and intention has to be interpreted with caution, because univariate analyses showed the opposite relationship, which indicates a possible suppressor-effect. This suppressor-effect was probably caused by the high correlation between decisional conflict and habit (*r* = .49; *p* < .001).

#### Daughters

The unique contribution of HPV vaccination uptake (first step) to the explained variance of vaccination intention was large and significant (R^2^ = .36, *p* < .001). The additional contribution of sample (second step) and socio-demographic variables (third step) to the explained variance of vaccination intention was not significant (ΔR^2^ = .00, *p* = .69; and ΔR^2^ = .02, *p* = .17, respectively), whereas the additional contribution of social-psychological variables (fourth step) was large and significant (ΔR^2^ = .34; *p* < .001). Attitude, beliefs, subjective norms and habit strength and risk perception (perceived susceptibility of getting cervical cancer without having received the HPV vaccination) significantly and uniquely contributed to the explanation of HPV vaccination intentions.

#### Stability

The stability of these models was tested on 25% of the total sample of mothers (*n* = 220) and daughters (*n* = 160). Results have to be interpreted with caution for country of birth because of the small percentage of participants born outside the Netherlands (13% of mothers and 5% of daughters). The outcomes of these stability tests are also presented in Tables 3 (mothers) and 4 (daughters).

#### Mothers

Country of birth and anticipated regret no longer significantly contributed to the prediction of HPV vaccination intention. Attitude, beliefs, subjective norms, habit strength and decisional conflict (certainty) appeared to be stable factors since they contributed to the explained variance in both the 25% and 75% samples.

#### Daughters

Beliefs, habit strength and risk perception no longer significantly contributed to the explained variance of HPV vaccination intention. Anticipated regret when refraining from vaccination significantly and positively contributed to HPV vaccination intention for the 25% sample, though it did not for the 75% sample. Attitude and subjective norms appeared stable since they significantly entered the equation in both samples.

#### Generalizability

The generalizability of determinants of HPV vaccination intention was explored by replicating the final regression model found for the 75% samples in the remaining 25% samples. The explained variance of the final models remained high for the 25% samples (R^2^ = .80 for mothers, R^2^ = .72 for daughters). This means that the final models found for the 75% samples appeared to predict to a large extent the vaccination intention of the mothers and daughters from the total population.

### Influential elements of the HPV vaccination intention determinants

More detailed insight into the most relevant determinants might be helpful for defining concrete objectives for designing interventions to promote HPV vaccination uptake. These analyses focused on the relative influence of individual items of the most relevant determinants from the final regression models (i.e., beliefs, subjective norms and habit for both mothers and daughters, and decisional conflict for mothers). Analyses for attitude were not conducted since the items on this scale were considered too general in nature to have practical relevance in this respect.

#### Beliefs

The following beliefs significantly (all *p* < .001 unless mentioned) correlated with the HPV vaccination intention of both mothers and daughters (The scales differ for positive (−3 = completely disagree to 3 = completely agree) and negative (−3 = completely agree to 3 = completely disagree) beliefs (see Table 1 for details). A higher score represents a more positive opinion about HPV vaccination): “If the government offers the vaccination, I assume it will be safe” (*r*Mother (*r*M) = .65 and *r*Daughter (*r*D) = .57); “The HPV vaccination was only introduced because the pharmaceutical industry will earn a lot of money from it” (*r*M = .60 and *r*D = .40); “Our government is showing responsibility for the health of the Dutch population by introducing the HPV vaccination” (*r*M = .60 and D*r* = .24); “There is too little known about whether the HPV vaccination effectively protects against cervical cancer” (*r*M = .59 and *r*D = .53); “There is too little known about the detrimental side effects of the HPV vaccination” (*r*M = .57 and *r*D = .42); “My daughter is too young to receive the HPV vaccination” (*r*M = .57 and *r*D = .40); and “My daughter does not need to get the HPV vaccination if she is already sexually active” (*r*M = .19; *r*D **=** .48, *p* = .001). The belief that a daughter does not need the vaccination because she is not yet sexually active positively and significantly correlated with the mothers’ HPV vaccination intentions (*r* = .63), whereas this relationship was not significant among daughters (*r* = .10; *p* = .03). The belief that it is inappropriate that the daughter has to pay for the HPV vaccination if she decides to get the vaccination at a higher age was positively and significantly related to the HPV vaccination intention of daughters (*r* = .23), while it was not significantly related to the intention of mothers (*r* = .05; *p* = .21).

#### Subjective norms

Mothers and daughters were significantly more likely to vaccinate against HPV when they expected a positive influence from their friends (*r*M = .58 and *r*D = .39), general practitioners (*r*M = .52 and *r*D = .42), physicians or nurses from the municipal health service (*r*M = .46 and *r*D = .41) and the Ministry of Health (*r*M = .42 and *r*D = .29; all *p* < .001). This also applied to mothers when they expected a positive influence from their partners (*r* = .80), daughters (*r* = .76) and parents (*r* = .66), and to daughters when they expected a positive influence from their mothers (*r* = .71) and fathers (*r* = .62; all *p* < .001).

#### Habit strength

Mothers and daughters were more inclined to accept the HPV vaccination when they perceived it as an automatic event (*r*M = .70 and *r*D = .61), without much thinking (*r*M = .47 and *r*D = .35; all *p* < .001).

#### Decisional conflict (certainty)

Decisional conflict (certainty) significantly contributed to the mothers’ HPV vaccination intention. Mothers were more likely to let their daughter receive the HPV vaccination if they perceived the decision as relatively easy to make (*r* = .22; *p* < .001). The item “As regards the HPV vaccination, I was clear about the best choice for my daughter” was borderline significant (*r* = .09; *p* = .01); mothers seemed more likely to let their daughter receive the HPV vaccination if they agreed with this statement. The other item on this scale was not significantly associated with the mothers’ HPV vaccination intentions: “As regards the HPV vaccination, I felt sure about what to choose” (*r* = .04; *p* = .28).

## Discussion

This paper presents empirically tested the social-psychological determinants of the HPV vaccination intention among girls and their mothers who received an invitation for the first Dutch catch-up campaign in 2009. This is one of the first studies to examine these determinants among participants who had actually made the decision about the HPV vaccination in the preceding year. Our results into determinants of the HPV vaccination decision confirm results from previous research that was performed before or after implementation (i.e., when girls and parents did not have to make the decision in the immediate future). Another insight of this study is that we combined various predictors from social-psychological research used in previous studies in one comprehensive set. As such, we were able to demonstrate which predictors of the set proved to be a sufficient, stable and generalizable combination. This is also one of the first studies that examined the contribution of these determinants to the explained variance of the HPV vaccination decision. This study revealed that HPV vaccination uptake made the largest contribution to the explained variance of HPV vaccination intention. We also found that social-psychological factors were far more important than socio-demographic factors in explaining the HPV vaccination intention. Moreover, social-psychological factors appeared to predict to a considerable extent the HPV vaccination intention of mothers and daughters from the total population.

HPV vaccination uptake made the largest contribution to the explained variance of HPV vaccination intention (47% among mothers, 36% among daughters). It is a common finding that past behavior is the best predictor of future behavior [28]. Secondary analyses revealed that exclusion of HPV vaccination uptake from the regression model did not decrease the prediction of HPV vaccination intention (mothers: R^2^ = .83 excluding and R^2^ = .84 including HPV vaccination intake; daughters: R^2^ = .73 including and excluding HPV vaccination intake). In addition, secondary analyses showed that social-psychological variables explained more than two-thirds of the variance in HPV vaccination intention when HPV vaccination uptake was excluded from the model (mothers: ΔR^2^ = .80; daughters: ΔR^2^ = .69). These results indicate that our final regression model was sufficient and contained all important social-psychological determinants [28].

The contribution of social demographic variables to the explained variance of the HPV vaccination intention appeared small (≤ 5%) and inconsistent. In contrast, the contribution of social-psychological determinants to the explained variance of the HPV vaccination intention was considerable (35% among mothers, 34% among daughters). This was an important finding, because the implications for future HPV vaccination communication are best found through these ‘modifiable’ social-psychological determinants of the HPV vaccination decision. Social-psychological factors that were not related to the HPV vaccination intention were, for example past cancer experiences and knowledge. With regard to past cancer experience, results of the present study confirm outcomes from a systematic review on HPV vaccination intention [12] and a study on other vaccinations [44]. These results reject the hypothesis that people are less willing to comply with national immunization programs because they are not as confronted with the seriousness of the target diseases as former generations, just because of the successes of these programs. In fact, almost all (>75%) study participants knew someone from their close environment who has or has had cancer (Tables 3 and 4). The absence of a relationship between knowledge and HPV vaccination intentions also confirms the results found by others [12,26,44-46]. This may also be attributed to the fact that knowledge gaps hardly existed; the percentage of correct answers was high, especially among mothers (≥ 70%).

Attitude, beliefs and subjective norms were the social-psychological factors that appeared to be most strongly and consistently related to HPV vaccination intentions. Attitude played the most prominent role in the explanation of the HPV vaccination intention, confirming previous findings [16,18,19,47]. The most dominant attitude-based beliefs were beliefs about the safety and effectiveness of the HPV vaccine, sexual maturation and optimism (misplaced or not) about future sexual risk taking, economic profit for the pharmaceutical industry, and trust in the government’s policies with respect to prevention of infectious diseases. Many relationships found in this study confirm others’ findings, such as beliefs about the safety and effectiveness of the HPV vaccine [12,47], sexual maturation and perceived future risk taking [12,47] and trust in the government’s prevention policies [19,21].

With regard to subjective norms, the influence of important others on HPV vaccination intention was most dominant for close family members (mother, father, and daughter). These results are similar to other studies about HPV vaccination [27] or the intention to take vaccinations in general [44]. Future communication strategies should therefore primarily be focused on these close family members. Aside from the expected social rewards, the mothers’ parents, friends, the general practitioner, and the physician or nurse from the regional health service also played a role in the decision making process. The impact of the subjective norms of physicians on HPV vaccination decision making was also previously found [12,19,20]. Because there was a lot of public debate about the HPV vaccination among physicians before the onset of the first catch-up campaign [48], future communication strategies should also target physicians as important party to make them aware of their influence on HPV vaccination decision making by mothers and daughters.

Social-psychological factors that were also, but less consistently, related to HPV vaccination intention, were risk perception, anticipated regret, and habit strength. As for risk perception, daughters were more inclined to accept the vaccination if they felt a higher perceived susceptibility to cervical cancer without the HPV vaccination. This was also found by others [12,16,17,49]. Mothers were also more inclined to accept the HPV vaccination for their daughter when they anticipated more feelings of regret if their daughters received no vaccination and developed cervical cancer later in life. Mothers who anticipated more feelings of regret if their daughters contracted a serious illness because of the HPV vaccination had a lower intention to let their daughters get the HPV vaccination. Secondary analyses indicated that the respondents anticipated stronger feelings of regret towards illness without having received the vaccination than after having received the vaccination. The importance of anticipated regret confirms what was found by other studies on HPV vaccination decision making [50] and on the intention of child vaccinations in general [44].

With regard to habit, mothers had higher intentions to let their daughters receive the HPV vaccination if they perceived getting the vaccination as something they did automatically or without thinking. This is probably caused by past confidence in the NIP. However, people who perceive the HPV vaccination as something one does automatically, without much thinking, may be more susceptible to counterarguments compared to deliberate decision makers [44]. They should therefore be better prepared for possible confrontation with those counterarguments [44].

### Strengths

One important strength of this study is the generalizability of the study results: the social-psychological factors appeared to predict to a large extent the vaccination intention of mothers and daughters from the total population. Another strength was that our final regression model contained all important social-psychological determinants [28]. Also, the total study sample was representative for the Dutch population with regard to educational level [41], country of birth [42] and religion [43]. This was a notable strength, because part of our sample was recruited via an online panel and the representativeness of online panels may be limited [51]. Another strength was that the HPV vaccination uptake among study participants corresponded to the national HPV vaccination coverage in 2009 [52]. In addition, self-reported vaccination uptake appeared to be a reliable measure for the objective vaccination uptake from the national vaccination register; there was a high percentage of agreement between them (>90% of mothers and daughters). Our choice to use the mothers to represent the parents’ HPV vaccination opinion appeared to be adequate, since almost all participants (>90% of mothers and daughters) indicated that there was no difference in opinion between the parents.

### Limitations

The present study had some limitations that are worth mentioning. First, it was based on a cross-sectional design, which makes it impossible to draw definite conclusions about causality of the associations found. Second, the response rate among subjects from the Praeventis sample was very low (7%). A low response rate (25%) was also found in another study which recruited parents through Praeventis to examine their attitude towards universal vaccination against hepatitis B [53]. The low response rate in the present study could be explained by the use of online questionnaires, because using such questionnaires results in lower response rates than using other types of questionnaires [54,55]. In addition, the use of a written invitation to recruit participants could have caused the low response rate, because such an invitation result in lower response rates than an e-mail invitation in studies using online questionnaires [54]. Besides, participants recruited by Praeventis received no reward for their participation in the study, whereas participants from the online panel did. Although the reward was small, it can increase response rates in questionnaire research [56]. Also, the low response rate among participants from Praeventis compared to participants from the online panel could be explained by the differences in the nature between the samples. Participants from an online panel chose to be part of that panel and are therefore self-selected. This does not apply to participants from Praeventis. Finally, response rates are declining recently [57], probably due to over-surveying. The usefulness of information gained from a sample with such a low response rate is questionable. However, we have reason to believe that the results of this sample are useful, because the total study sample was representative for the Dutch population (see also “Strengths”).

### Implications

#### Future research

We recommend a longitudinal research approach in order to confirm the causality of the associations between social-psychological factors and HPV vaccination behavior.

#### Future communication strategies

We recommend future communication addressing social-psychological variables that appear to be related to HPV vaccination (i.e. attitude, beliefs, subjective norms, habit, anticipated regret and perceived susceptibility). For example, future communication could address important beliefs about the safety of the vaccine, could reduce unrealistic optimism about the expected monogamy of the daughter and could emphasize the importance of receiving the HPV vaccination before daughters become sexually active. Furthermore, future communication strategies about the HPV vaccination should be targeted at daughters and their parents. Both parents appeared to play the most important role in mothers’ and daughters’ vaccination decision making. In addition, we recommend maintaining or improving confidence in authorities involved in the HPV vaccination, for example, by preventing conflict of interest with the pharmaceutical industry, or by communicating about safety as well as risks of the HPV vaccination. Finally, a tailored communication approach should be considered, because it has already been successfully applied to a variety of health-related behaviors, especially in targeting social-psychological variables also found in the present study [58,59].

## Conclusions

This is one of the first studies to examine determinants of the HPV vaccination intention among participants who had actually made the decision about the HPV vaccination in the preceding year. The study revealed that social-psychological factors were far more important than socio-demographic factors in explaining HPV vaccination intention. Attitude, beliefs, subjective norms and habit strength were significantly associated with both the mothers’ and the daughters’ HPV vaccination intentions. Moreover, the generalizability of the study results was adequate. Future communication strategies targeting HPV vaccination uptake should address these determinants.

## Competing interests

The authors declare that they have no competing interests.

## Authors’ contribution

HMVK and MF developed and executed the study, with TWGMP, WO, RACR and JVS advising on its development. ED advised on the conceptions of data collection and executed parts of the data analyses. HMVK and TWGMP significantly contributed to the writing of the manuscript, while WO, RACR, MF, JVS and ED were involved in revising it. All authors have seen and approved of the version to be published.

## Pre-publication history

The pre-publication history for this paper can be accessed here:

http://www.biomedcentral.com/1471-2458/13/111/prepub
